# Association between ambient temperatures and injuries: a time series analysis using emergency ambulance dispatches in Chongqing, China

**DOI:** 10.1265/ehpm.22-00224

**Published:** 2023-05-11

**Authors:** Zhi-Yi Chen, Hui Hu, Jun Yang, Dian-Guo Xing, Xin-Yi Deng, Yang Zou, Ying He, Sai-Juan Chen, Qiu-Ting Wang, Yun-Yi An, Ying Chen, Hua Liu, Wei-Jie Tan, Xin-Yun Zhou, Yan Zhang

**Affiliations:** 1 School of Public Health, Research Center for Medicine and Social Development, Innovation Center for Social Risk Governance in Health, Research Center for Public Health Security, Chongqing Medical University, No.61 Middle University Town Road, Shapingba District, Chongqing 400016, China; 2 Department of Traumatology, Chongqing University Central Hospital, Chongqing Emergency Medical Center, No.1 Jiankang Road, Yuzhong District, Chongqing, China; 3 Office of Health Emergency, Chongqing Municipal Health Commission, No.6 Qilong Road, Yubei District, Chongqing 401147, China

**Keywords:** Extreme temperatures, Injury emergency ambulance dispatches (IEADs), Distributed lagged nonlinear model (DLNM), Attributable burden

## Abstract

**Background:**

Global warming and increasing extreme weather have become a severe problem in recent years, posing a significant threat to human health worldwide. Research exploring the link between injury as one of the leading causes of death globally and ambient temperature was lacking. Based on the hourly injury emergency ambulance dispatch (IEAD) records from 2019–2021 in the main urban area of Chongqing, this study explored the role of temperature extremes on the pathogenesis of injury by different mechanisms and identified sensitive populations for different mechanisms of injury.

**Methods:**

In this study, we collected hourly injury emergency ambulance dispatch (IEAD) records from Chongqing Emergency Dispatch Center in the main urban area of Chongqing from 2019 to 2021, and used a distributed lagged nonlinear model (DLNM) with quasi-Poisson distribution to evaluate the association between ambient temperature and IEADs. And the stratified analysis was performed by gender, age and different injury mechanisms to identify susceptible groups. Finally, the attributable burden of ambient extreme temperatures was also investigated.

**Results:**

The risk for total IEADs increased significantly at high temperature (32 °C) compared with optimal temperature (9 °C) (CRR: 1.210; 95%CI[1.127,1.300]). The risks of traffic accident injury (CRR: 1.346; 95%CI[1.167,1.552]), beating injury (CRR: 1.508; 95%CI[1.165,1.952]), fall-height injury (CRR: 1.871; 95%CI[1.196–2.926]) and injury of sharp penetration (CRR: 2.112; 95%CI[1.388–3.213]) were significantly increased. At low temperature (7 °C), the risk of fall injury (CRR: 1.220; 95% CI [1.063,1.400]) increased significantly. Lag for 24 hours at extreme low temperature (5 °C), the risk of 18–45 years (RR: 1.016; 95%CI[1.009,1.024]) and over 60 years of age (RR: 1.019; 95%CI[1.011,1.025]) increased significantly. The effect of 0 h delay in extreme high temperature (36 °C) on males aged 18–45 years (RR: 1.115; 95%CI[1.071,1.162]) and 46–59 years (RR: 1.069; 95%CI[1.023,1.115]) had significant impact on injury risk.

**Conclusions:**

This study showed that ambient temperature was significantly related to the risk of injury, and different mechanisms of injury were affected differently by extreme temperature. The increasing risk of traffic accident injury, beating injury, fall-height injury and sharp penetrating injury was associated with extreme heat, while fall injury was associated with extreme cold. The risk of injury in high temperature environment was mainly concentrated in males and young adults. The results of this study can help to identify the sensitive population with different injury mechanisms in extreme temperature environment, and provide reference for public health emergency departments to respond to relevant strategies in extreme temperature environment to minimize the potential risk to the public.

**Supplementary information:**

The online version contains supplementary material available at https://doi.org/10.1265/ehpm.22-00224.

## 1. Introduction

Global warming and increasing extreme weather have become more and more serious in recent years. According to the data of the Sixth Assessment Report released by IPCC, global warming will increase 1.5 °C during 2030 and 2052. In the future, the surface temperature will continue to rise, and extreme weather will be more frequent and last longer [1], which poses a great threat to human health around the world [2, 3]. Therefore, it is necessary to assess the negative health effects of extreme temperatures to provide a scientific basis for the development of appropriate public health policies and interventions.

Injury is one of the leading causes of death in the world, killing about 5 million people every year, accounting for 9% of all deaths, and about a quarter of these occur in low and middle-income countries [4]. As the largest developing country, about 730,000 people died for injuries in China in 2017, accounting for 7% of the total deaths [5], bringing a significant disease burden to the society. In recent years, the exploration of factors affecting the incidence of injury has gradually begun, but these studies mainly explored the impact of demographic [6, 7] and economic [8] factors on the incidence of injury. Climatic factors, such as extreme temperature, can also increase the risk of injury through the impact on the external environment and the physical and psychological impact on the human body [9, 10]. However, there were few studies in this area and the results were different. For example, Song et al. [11] only found a significant effect of high temperature on traffic accidents, while Zhao et al. [12] only found a significant effect of low temperature on traffic accidents. In the studies of Liang et al. [13] and Hussain et al. [14], it was found that extreme temperature had a significant effect on fall injuries, while Lin et al. [15] did not find such a relationship. The differences in the above studies might result from differences in climatic conditions and population characteristics in different regions. In addition, most of the previous studies used a minimum time scale of days, but the effect of extreme temperature on injury onset was mostly an immediate effect or a lag effect of 1–3 days [16, 17]. Therefore, this paper shortened the time scale to hours in order to more accurately capture the immediate and lagged effects of extreme temperature on injury onset.

The main urban area of Chongqing is located in the southwest of China, which is dominated by mountainous terrain with rich geomorphologic changes and difficult travel for people, so the risk of injury is high. Therefore, based on the hourly IEADs in the main urban area of Chongqing from 2019 to 2021, this study used distributed lag nonlinear model (DLNM) to evaluate the effects of extreme temperatures on injury incidence of different mechanisms. At the same time, population stratified analysis was conducted to explore the effects of extreme temperatures on injury incidence in populations with different characteristics. The results help to provide a reference for the implementation of more rational injury interventions and the deployment of injury emergency resources.

## 2. Materials and methods

### 2.1 Study area

The investigation of this study was carried out in the main urban area of Chongqing, including Yuzhong District, Jiangbei District, Nanan District, Jiulongpo District, Shapingba District, Dadukou District, Yubei District and Banan District. Chongqing has a subtropical monsoon humid climate. The four seasons can be clearly divided by the climate temperature method. The average precipitation is relatively abundant, mostly concentrated from May to September, and the annual average relative humidity is mostly 70%–80%, which belongs to the high humidity area in China. The average resident population of the study area was about 9.51 million during 2019–2021, and the emergency ambulance dispatches during the study period were 414190.

### 2.2 Data collection

#### 2.2.1 Injury Emergency Ambulance Dispatch (IEAD)

Chongqing Emergency Dispatch Center provided hourly IEAD records from January 1, 2019 to December 31, 2021. IEAD records pertinent information about the patient, including time, region, age, gender, symptoms, and initial diagnosis. The initial diagnosis was made by the physician prior to the patient’s arrival at the hospital and was recorded in a nonuniform descriptive text, and the IEAD records were classified according to the International Classification of Diseases, 10th Revision (ICD-10). IEAD records without gender and age were excluded from our stratified analysis.

#### 2.2.2 Environmental data

Meteorological data were obtained from the National Climatic Data Center (NCDC) and included hourly mean temperatures (°C). Pollution data were obtained from of the China National Environmental Monitoring Station and included particulate matter with an aerodynamic diameter of less than 10 µm (PM_10_: µg/m^3^) and ozone (O_3_: µg/m^3^).

### 2.3 Data analysis

We employed quasi-Poisson distributed lagged nonlinear model (DLNM) [18–20] to remove the nonlinear and lagged effects of IEADs due to extreme temperatures by controlling hourly average temperature, long-term trends, hours of the day, day of the week, and holidays [21–23]. The model used was as follows:

$$\begin{array}{rl} \text{Log}(\text{E}({\text{Y}}_{\text{t}})) & =\alpha +\beta_{1}{\text{temp}}_{\text{t,l}}+\beta_{2}\text{NS}(\text{time},7)\\ & \;+\beta_{3}\text{NS}(\text{hod},6)+\beta_{4}\text{DOW}+\beta_{5}\text{Holiday} \end{array}$$

Where E (Y_t_) was the expected amount of IEAD at t hours, α was the intercept, and β_1_–β_5_ was the coefficient of the regression model. Temp_t,l_ was a cross-base for temperature exposure and lag time, using a natural cubic spline with 4 degrees of freedom (df) to represent temperature and 4 df to represent lag to capture nonlinear temperature and lag effects, respectively. Time represented the confounding effect of long-term and seasonal trends, controlled by a natural cubic spline with 7 df. Hour was the number of hours in a day and was represented by a natural cubic spline function with 6 df. The dfs were chosen according to the minimum of Akaike’s Information Criterion for quasi-Poisson (quasi-AIC). DOW indicated the day of the week, and holiday indicated whether it was a holiday.

After the model was constructed, the overall cumulative and single-lag associations of the temperature range with IEADs for different mechanisms and populations were plotted and evaluated. The optimal temperature (OT) was set as the temperature with minimum IEADs risk in different temperature exposure situations, which was used as a reference. When there was no minimum IEADs risk temperature, we assessed the risk of extreme temperature (5_th_ and 95_th_). At last, the effect of low temperature (1_th_) and high temperature (99_th_) on the relative risk (RR) of injury for different populations and mechanisms were evaluated.

According to the relationship between the temperature and IEAD, the attribution scores of IEADs to low temperatures (<5_th_), high temperatures (>95_th_) and overall temperature were calculated using the method proposed by Gasparrini et al. [24, 25]. The 95% confidence intervals (95%CI) of attribution scores and attribution adjustment quantity were obtained by Monte Carlo simulation.

### 2.4 Sensitivity analysis

In this study, some sensitivity analyses were conducted, including: (1) further adding pollution factors PM_10_ and O_3_ to the model (Fig. S1); (2) changing df of time (df = 5,6,7); (3) changing df of hour (df = 5,6,7); (5) changing the maximum lag (48, 60, 72) (Table S1).

All statistical analyses were performed using RStudio (package: dlnm).

## 3. Result

### 3.1 Descriptive analysis

**Fig. 1:**
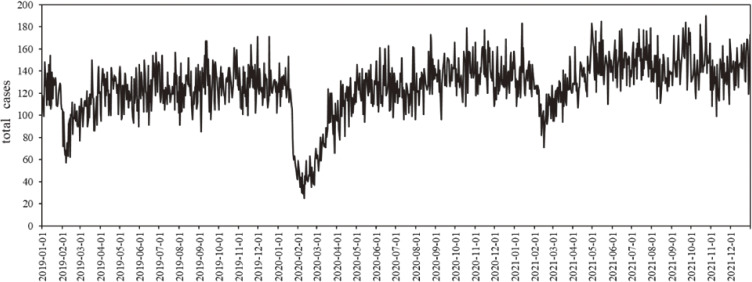
Time series distribution of IEADs in the main urban area of Chongqing from 2019 to 2021

Table 1 summarized the descriptive analysis of the hourly IEADs by different mechanisms of injury and environment variables in the main urban area of Chongqing. This study experienced 26304 hours in the main urban area of Chongqing, and a total of 169121 IEAD records were collected. The average number of IEAD per hour was 6.43 ± 3.77. The ratio of male to female IEAD was approximately 2:1, and 70% of IEADs were concentrated in those aged 18 to 45 years and over 60 years. The main injury mechanisms were fall (30.07%) and traffic accident (29.13%), and the average number of ambulance dispatches per hour was 1.93 ± 1.89 and 1.87 ± 1.84, respectively. Figure 1 showed the time series distribution of IEADs in the main urban area of Chongqing from 2019 to 2021. Among the environmental factors, the average hourly temperature, PM_10_ and O_3_ were 18.76 ± 8.03 (°C), 58.33 ± 31.54 (µg/m^3^) and 41.19 ± 41.49 (µg/m^3^), respectively (Table 1). Environmental factors were significantly correlated with IEADs (p < 0.001).

**Table 1:** Descriptive analysis of IEADs and environment factors.

	**Subgroups**	**Percent%**	**Mean(hourly) ± Sd**	**Min**	**P25**	**P50**	**P75**	**Max**
**All**			6.43 ± 3.77	0	4	6	9	33
**Mechanism of Injuries**								
	Fall	30.07	1.93 ± 1.89	0	0	1	3	14
	Traffic accident	29.13	1.87 ± 1.84	0	0	1	3	15
	Beat	8.55	0.55 ± 0.88	0	0	0	1	9
	Sharp	3.05	0.20 ± 0.50	0	0	0	0	5
	Fall-Height	2.82	0.18 ± 0.50	0	0	0	0	6
	Else	26.38	1.70 ± 1.91	0	0	1	3	29
**Environment factors**								
	Mean temperature (°C)		18.76 ± 8.03	−1.00	11.90	18.30	25.00	40.70
	PM_10_ (µg/m^3^)		58.33 ± 31.54	9.58	35.25	51.08	74.33	305.45
	O_3_ (µg/m^3^)		41.19 ± 41.49	2.75	13.44	27.42	51.76	319.45

### 3.2 Relationship between ambient temperature and IEADs

**Fig. 2:**
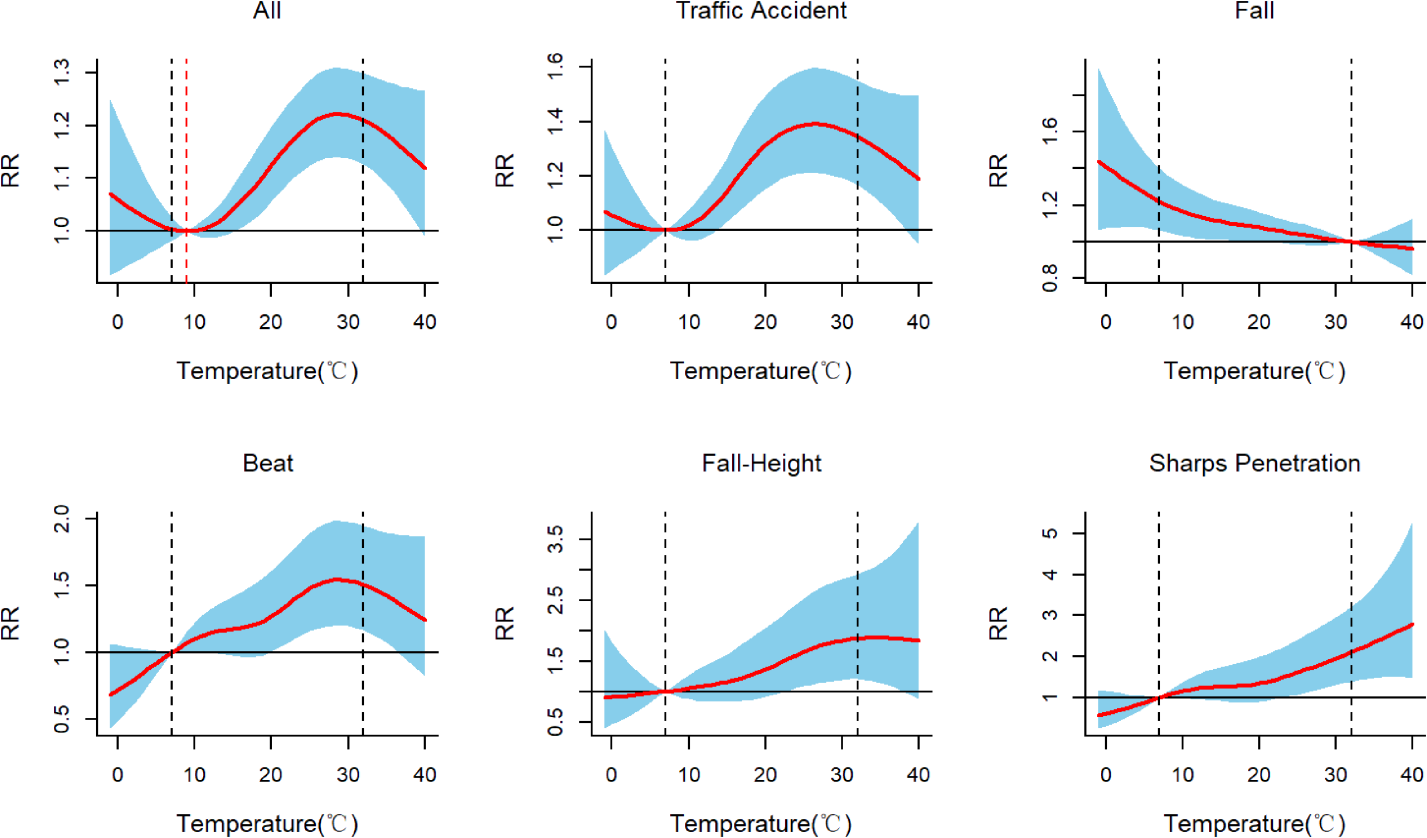
Lag results of IEADs exposed to temperatures (blue area: 95% CI of the RR).

The Fig. 2 showed the response association between the overall cumulative exposure and the associated temperature distribution for different mechanisms of IEADs in the main urban area of Chongqing with a 72-hour lag. In the high temperature environment (32 °C), compared with the OT (9 °C), the risks of all IEADs was significantly increased (CRR: 1.210; 95%CI[1.127,1.300]). Compared with 7 °C, the risk of traffic accident injury (CRR: 1.346; 95%CI[1.167,1.552]), beating injury (CRR: 1.508; 95%CI[1.165,1.952]), fall-height injury (CRR: 1.871; 95%CI[1.196,2.926]) and sharps penetration injury (CRR: 2.112; 95%CI[1.388,3.213]) were significantly increased in the high temperature environment (32 °C). In the low temperature environment (7 °C), compared with 32 °C, the risk of fall injury was significantly increased at 7 °C (CRR: 1.220; 95%CI[1.063–1.400]). For the analysis in different populations, it was found that the male population (CRR: 1.266; 95%CI[1.105–1.451]) and those aged 18–45 years (CRR: 1.370; 95%CI[1.166–1.609]) were significantly affected by high temperatures (Fig. S2).

### 3.3 Effect of lagged effect

**Fig. 3:**
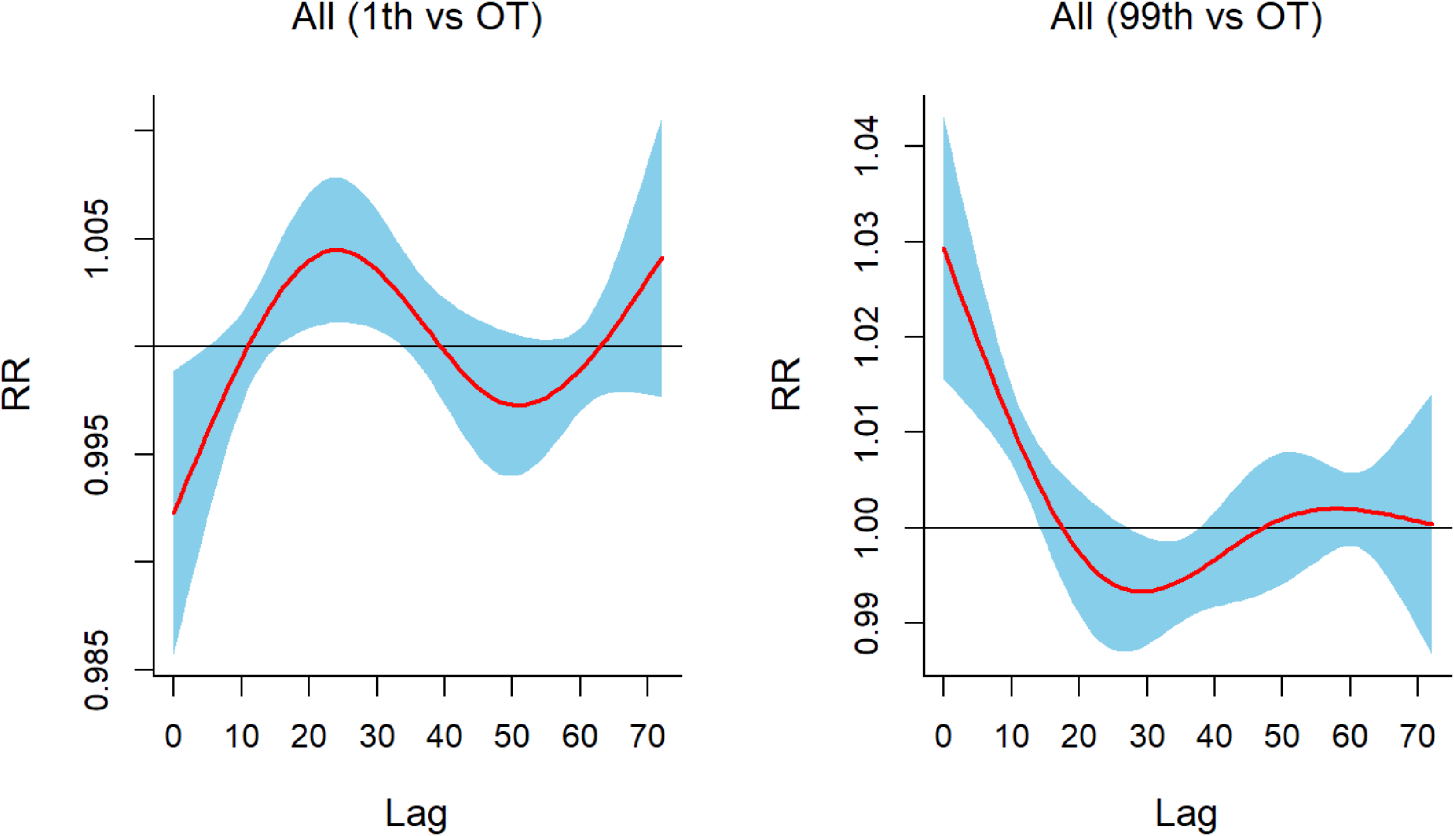
Lag response association between extreme temperatures (Left: 5 °C, 1th percentile temperature; Right: 36 °C, 99th percentile temperature)

Figure 3 showed the lag response of IEAD in the main urban area of Chongqing under conditions of extreme low temperature (5 °C: 1st percentile temperature) and extreme high temperature (36 °C: 99th percentile temperature). In the overall population, the RR of IEAD peaked at a lag of 24 hours under extreme low temperature, and extreme heat had a significant immediate effect on IEAD. The lags of different populations in extreme temperature environments were shown in Fig. S3–S4.

**Fig. 4:**
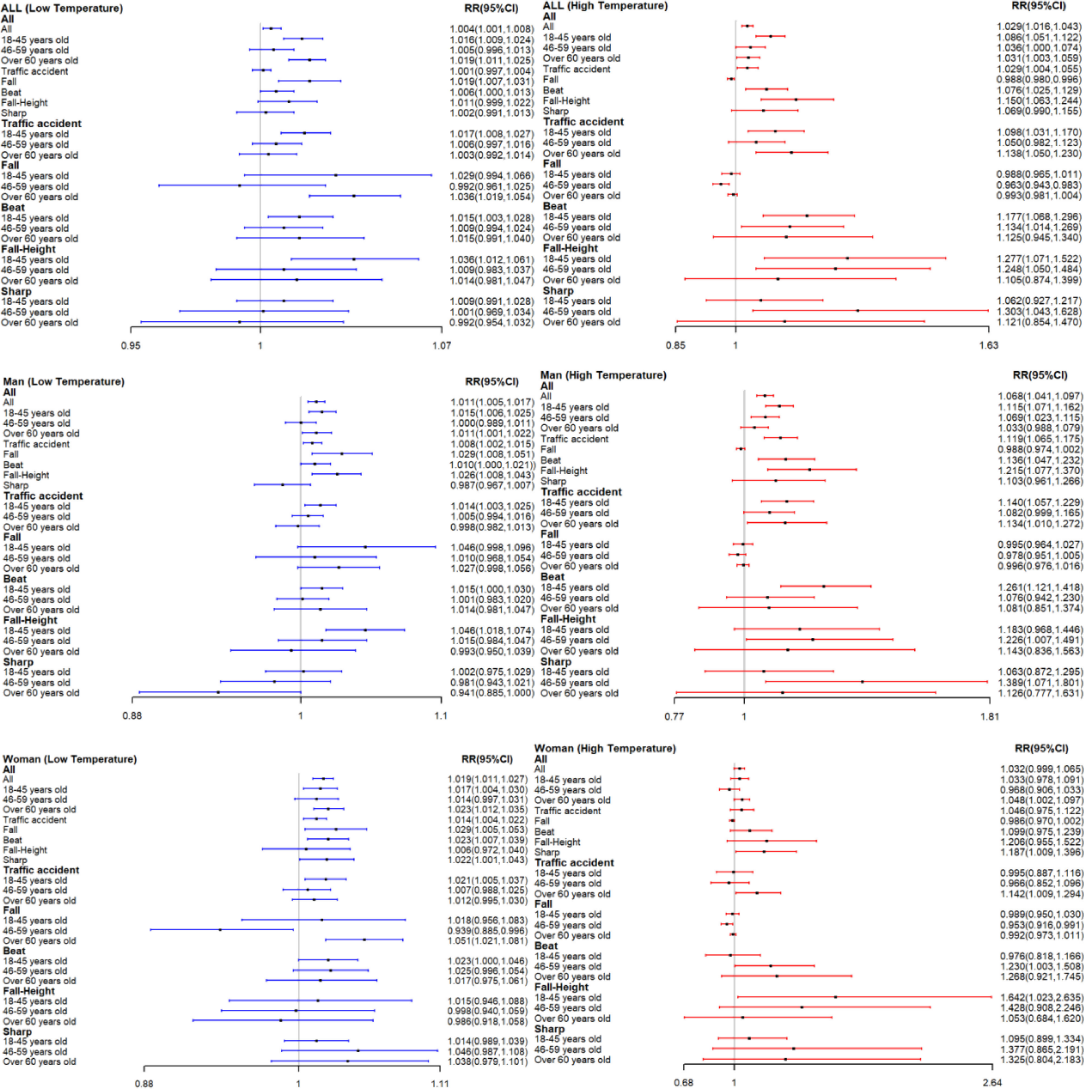
Lag effects of 1_th_ percentile temperature (5 °C) and 99_th_ percentile temperature (36 °C) for IEADs.

Figure 4 showed the lag effects of extreme high temperature (36 °C: 99th percentile temperature) and extreme low temperature (5 °C: 1st percentile temperature) in the main urban area of Chongqing on the IEADs in subgroups during the study period. Extreme low temperature was significantly associated with an increasing risk of IEAD, especially for ages 18–45 years (RR: 1.016; 95%CI[1.009,1.024]) and people over 60 years old (RR: 1.019; 95%CI[1.011,1.025]). In terms of injury mechanism, extreme cold was significantly associated with an increased risk of fall injury (RR: 1.019; 95%CI[1.007,1.031]), especially in women over 60 years old (RR: 1.051; 95%CI[1.021,1.081]). And extreme cold was significantly associated with an increase in fall-height injuries among men aged 18–45 years (RR: 1.046; 95%CI[1.018,1.074]). In addition, extreme heat was significantly associated with an increasing risk of IEAD, especially for men aged 18–45 years (RR: 1.115; 95%CI [1.071,1.162]) and 46–59 years (RR: 1.069; 95%CI[1.023,1.115]). In terms of injury mechanism, extreme heat was significantly associated with an increase in traffic accident injury, especially for people over 60 years old (RR: 1.138; 95%CI[1.050,1.230]) and men aged 18–45 years (RR: 1.140; 95%CI[1.057,1.229]). Beating injuries were significantly increased in extreme heat environment, especially in males aged 18–45 years (RR: 1.261; 95%CI[1.121,1.418]) and women aged 46–59 years (RR: 1.230; 95%CI[1.003,1.508]). Extreme heat was significantly associated with an increase in fall-height injury, especially among men aged 46–59 years (RR: 1.226; 95%CI[1.007,1.491]) and women aged 18–45 years (RR: 1.642; 95%CI[1.023,2.635]). And extreme high temperatures were significantly associated with an increased risk of sharp penetrations injury in men aged 46–59 years (RR: 1.389; 95% CI [1.071,1.801]).

### 3.4 Attributable risk

Table 2 showed the attributable numbers and fractions and 95% confidence interval (95%CI) of IEADs due to low temperature and high temperature using a backward perspective. A total of 9.293% (95%CI: 5.945%–12.829%) of the increase in IEADs was attributed to temperature during the study period, and 1.130% (95%CI: 0.662%–1.565%) of the increase in IEADs was attributed to high temperature (32 °C–40.7 °C). Traffic accident injuries (1.932%, 95%CI: 0.957%–2.805%), beating injuries (1.945%, 95%CI: 0.633%–3.137%), fall-height injuries (3.340%, 95%CI: 0.382%–5.502%) and sharp penetration injuries (5.438%, 95%CI: 2.531%–7.570%) were significantly attributed to high temperature. Low temperature significantly increased the incidence of fall injuries (1.507%, 95%CI: 0.622%–2.292%).

**Table 2:** The attributable numbers and fractions and 95%CI of IEADs due to extreme temperature.

**Mechanism of Injuries**	**Backward**	**Temperature**	**Low temperature**	**Hot temperature**
			interval:min(−1 °C)-5th(7 °C)	interval:95th(32 °C)-max(40.7 °C)
All	AF(%)	9.293(5.945,12.829)	0.097(−0.165,0.339)	1.130(0.662,1.565)
	AN	15715.68(9526.418,21712.720)	164.390(−262.032,584.488)	1911.195(1113.279,2666.792)
Traffic accident	AF(%)	17.700(9.70,24.91)	0.026(−0.179,0.217)	1.932(0.957,2.805)
	AN	8718.802(4757.103,12273.644)	12.788(−87.624,108.897)	951.873(521.260,1339.905)
Fall	AF(%)	8.469(2.118,14.002)	1.507(0.622,2.292)	−0.197(−0.383,−0.015)
	AN	4307.432(1156.328,7152.059)	766.559(304.605,1186.652)	−100.186(−195.136,−10.208)
Beat	AF(%)	20.83(5.537,31.792)	−0.256(−0.621,0.076)	1.945(0.633,3.137)
	AN	3012.571(996.203,4623.169)	−36.956(−87.656,11.799)	281.231(71.723,465.188)
Fall-Height	AF(%)	25.68(0.530,43.275)	0.097(−0.563,0.647)	3.340(0.382,5.502)
	AN	1222.991(51.330,2089.041)	4.616(−25.496,28.303)	159.089(29.687,256.406)
Sharp	AF(%)	29.21(4.821,45.916)	−0.411(−1.043,0.084)	5.438(2.531,7.570)
	AN	1508.777(225.441,2404.039)	−21.239(−50.438,3.340)	280.936(121.538,400.471)

## 4. Discussion

Based on the hourly dispatch data provided by ambulance institutions, this paper studied the association between the incidence of IEAD by different mechanisms and temperature in Chongqing from 2019 to 2021. The results showed that high temperature had a significant cumulative lag effect on the increase of all IEADs, and the male aged 18–59 years was significantly affected by high temperature. Different mechanisms of IEAD were affected differently by extreme temperature.

From 2019 to 2021, the number of IEAD in Chongqing was 169, 121, accounting for one third of the total ambulance dispatching, among which injuries caused by fall and traffic accident injuries accounted for the largest proportion. The finding of an immediate effect of hot weather on the increasing risk of IEAD followed similar findings in some previous studies [26, 27]. The attribution results also found that 1.130% (95%CI: 0.662%–1.565%) of the increase in IEAD was attributed to high temperature. And the risk of injury in men aged 18–59 years was significantly increased with high temperature. This might be because this group of people was the main labor force of social work, and they always needed to work in high temperature environments, so they were more vulnerable to external harm. However, there was a lag effect of low temperature on the increase of IEAD risk, which was more significant in people aged 18–45 years and over 60 years. This could be due to the increase in clothing and slippery conditions in cold weather, making it more difficult for people to travel and more likely to be injured.

This study found that the risk of traffic accident injuries was significantly increased in extreme heat environment, and similar conclusions were obtained by previous studies [28, 29]. This might be because in hot weather, the coefficient of friction between the tire and the road surface could be reduced, increasing the risk of traffic accidents [9]. In the subgroup study, we found that extreme heat was significantly associated with increased risk of traffic injury among males aged 18–45 years and people over 60 years. On the one hand, young and middle-aged men were the main group of drivers. Some experimental studies had found that ambient temperature was negatively correlated with biomarkers of serotonin [10, 30], suggesting that high ambient temperature might lead to serotonergic dysfunction, which was a sign of decreased decision-making ability and violent impulses [31]. And this might lead to irritability and impatience of drivers to increase the risk of traffic accidents. On the other hand, with the increase of age, various physiological functions of people began to degenerate, so the stride and speed of people over the age of 60 decreased significantly, and their perception function deteriorated, which made the elderly take longer to cross the road than the young, and they were more vulnerable to traffic accidents in hot summer [32]. Therefore, meteorological management departments should warn the public in advance of extremely high temperature weather, carry out relevant health knowledge propaganda, and try to avoid their high temperature stress driving. At the same time, the traffic department should start the high temperature plan of traffic accidents quickly to prevent the occurrence of traffic accidents as far as possible.

The study found that the risk of fall injury was significantly increased in cold environments, and there was a significant lag effect in the female over 60 years. On the one hand, low temperature was often accompanied by rain and snow, and the road was slippery [33]. In addition, the transmission speed of the human nervous system was slowed down under low temperature, and the flexibility of muscles and bones was reduced [34], which had a greater impact on the elderly with slow movement, leading to a significant increase in the risk of falls in the elderly. On the other hand, studies had shown that the incidence of osteoporosis in the elderly over 60 years old had reached more than 30%, and the incidence of female was much higher than that of male [35]. Therefore, these people were more likely to cause serious injuries after falling down, which increased the number of ambulance dispatches. It is suggested that relevant departments should lay anti-skid pavement in elderly activity venues and fitness trails, and give priority to rainwater clearing routes in combination with elderly activity routes planning [36].

This study found that the risk of beating and sharp penetrations injuries increased significantly in extreme heat environment, especially in men aged 18–45 years and men aged 46–59 years, respectively. This might be because people in Chongqing had a rich night life [37] and a popular spirits culture [38]. In the environment of high temperature and humidity, people preferred to go out at night to drink to relieve heat and dampness. However, excessive alcohol consumption could lead to cognitive impairment, as well as impairment of emotional processes and social cognition [39]. At the same time, exposure to high temperature also reduced brain serotonin [40], and the related effects of temperature on platelets also increased impulsive aggression in the population [41]. Therefore, the public security problem caused by drinking and rioting at night was also serious [42], which lead to an increase in the risk of beating injury and sharp penetration injury. The hidden danger of excessive drinking should be vigorously publicized to the public, and the density of patrol police should be strengthened to improve the level of public security management. On the other hand, previous studies had found that workers under high temperature had a higher risk of occupational injuries due to mechanical and manual operations [43]. This might be because extreme heat can lead to body fatigue, cognitive function and perceptual motor skills decline [44], so workers engaged in machinery related occupations were vulnerable to sharp instrument penetration injury due to negligence. Therefore, the relevant units should try to avoid the staff involved in machinery to work for a long time at high temperature, and should reasonably arrange their work and rest time.

We found that the risk of fall-height injury was significantly increased in young and middle-aged male population under extreme temperature, which might be because this group of people was the main group of people working high above the ground. On the one hand, extreme cold weather was often accompanied by rain and fog, which made the high-altitude workplace slippery and increases the work blind area, so the staff was more likely to fall from the height due to operational errors [45]. On the other hand, working in extreme high temperature was prone to dehydration and heat cringe, which damaged its ability to work safely [46] and increased the risk of falling from height. At present, the traditional safety management mode in the field of aerial works in China belonged to the post control [47]. In the future management model, we should try to avoid the risk of accidents brought by extreme weather in advance, improve the safety facilities in the workplace, and improve the safety awareness of workers. We also found that women aged 18–45 years were at increased risk of falling from height in extreme heat. This might be related to the negative effect of heat on people’s mood [48]. Some studies had found that high temperature had a greater negative impact on women’s mental health, which might increase the risk of suicide such as jumping off buildings or Bridges, resulting in an increase in falling injuries from high places [49, 50].

Our study had several strengths. First, our data were obtained from ambulance dispatch centers, which provided a better representation of the timeliness of the effect of extreme temperatures on the risk of injury than the outpatient or inpatient data used in previous studies. Secondly, this study evaluated the sensitivity of injury to extreme temperature in terms of gender and age with different mechanisms, which could help to identify the vulnerable population of injury in extreme temperature environment, so as to better inform the target population of the relevant hazards. And the results also provided reference for the public health emergency departments to respond to the relevant response strategies in extreme temperature environment, which reduced the potential risk to the public from extreme temperatures. Finally, as far as I know, this was the first study of the relationship between extreme ambient temperature and ambulance dispatches due to different causes of injury in southwest China. The results of this study could provide more evidence on the adaptation or susceptibility of different populations to climate change.

We also had several weaknesses. First, the study was only conducted in the main urban area of Chongqing, so there were still some limitations in the generalization of its results. Second, this study only used the information of fixed observation sites, but the temperature and pollutant exposure perceived by individuals might be different, so it can be further studied in the following study. Third, this study assumed that the moment of the call was the moment of the injury event and did not consider the possible delay in between. Finally, the initial diagnosis in this study was made by the scheduling physician prior to the patient’s arrival at the hospital and thus might have deviated from the final diagnosis.

## 5. Conclusion

This study showed that ambient temperature was significantly related to the risk of injury, and different mechanisms of injury were affected differently by extreme temperature. The increased risks of traffic accident injury, beating injury, fall-height injury and sharp penetrating injury was associated with extreme heat, while fall injury was associated with extreme cold. The risk of injury in high temperature environment was mainly concentrated in males and young adults. The results of this study can help to identify the sensitive population with different injury mechanisms in extreme temperature environment, and thus provide reference for public health emergency departments to respond to relevant strategies in extreme temperature environment to minimize the potential risk to the public.

## Abbreviations

IEADs: Injury emergency ambulance dispatches
DLNM: Distributed lagged nonlinear model
df: Degree of freedom
OT: The optimal temperature
CRR: Cumulative relative risk
RR: Relative risk
95%CI: 95% Confidence interval

## Supplementary information

**Additional file 1:** **Table S1** QAIC values adjusting for long-term trends, number of hours of freedom in a day and lag hours. **Figure S1** Overall cumulative exposure of IEADs in the main urban area of Chongqing and the related temperature distribution from 2019 to 2021 (PM_10_ added to the left and O_3_ added to the right). **Figure S2** Response association between the overall cumulative exposure and the associated temperature distribution for different populations of IEADs in the main urban area of Chongqing. **Figure S3** Lagged response association between extreme low temperature (5 °C: 1st percentile temperature) and extreme high temperature (36 °C: 99th percentile temperature) in different gender. **Figure S4** Lagged response association between extreme low temperature (5 °C: 1st percentile temperature) and extreme high temperature (36 °C: 99th percentile temperature) in different age groups.

## Declarations

### Availability of data and material

The data of IEADs cases analyzed in this study were obtained from the Chongqing Emergency Dispatch Center, but restrictions apply to the availability of these data, which were used under license for the current study, and so were not publicly available. Data were however available from the authors upon reasonable request and with permission of the above institutions.
