# Supplementary material for: Association between ambient temperatures and injuries: a time series analysis using emergency ambulance dispatches in Chongqing, China
Source: Environ Health Prev Med. 2023 May 11;28:28. doi: 10.1265/ehpm.22-00224 (PMC10188285; doi:10.1265/ehpm.22-00224)
Supplement: Supplementary file 1 — Additional file 1: Table S1 QAIC values adjusting for long-term trends, number of hours of freedom in a day and lag hours. Figure S1 Overall cumulative exposure of IEADs in the main urban area of Chongqing and the related temperature distribution from 2019 to 2021 (PM10 added to the left and O3 added to the right). Figure S2 Response association between the overall cumulative exposure and the associated temperature distribution for different populations of IEADs in the main urban area of Chongqing. Figure S3 Lagged response association between extreme low temperature (5 °C: 1st percentile temperature) and extreme high temperature (36 °C: 99th percentile temperature) in different gender. Figure S4 Lagged response association between extreme low temperature (5 °C: 1st percentile temperature) and extreme high temperature (36 °C: 99th percentile temperature) in different age groups. [file ehpm-28-028-s001.docx]

**Supplemental materials**

Table S1 QAIC values adjusting for long-term trends, number of hours of freedom in a day and lag hours

| Parameter | number of df | QAIC |
| --- | --- | --- |
| date | 5 | 109833.7 |
|  | 6 | 109388.7 |
|  | 7 | 109123.7 |
| hour | 5 | 109491.5 |
|  | 6 | 109123.7 |
|  | 7 | 109539.9 |
| lag hours | 48 | 115156.7 |
|  | 60 | 112051.9 |
|  | 72 | 109123.7 |


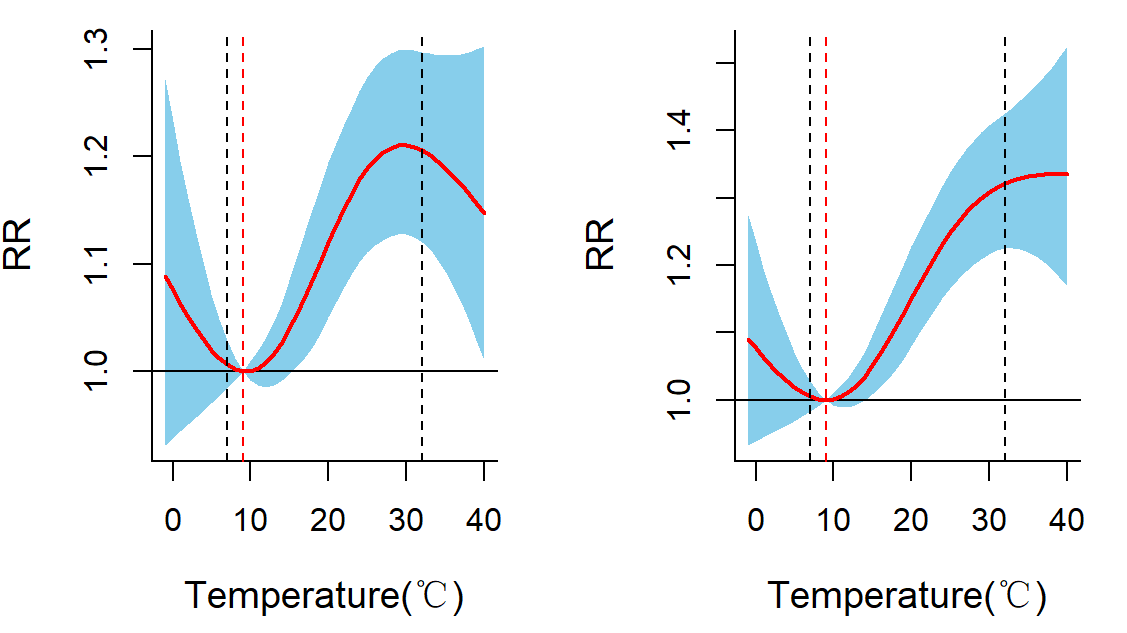


Figure S1 Overall cumulative exposure of IEADs in the main urban area of Chongqing and the related temperature distribution from 2019 to 2021(PM_10_ added to the left and O_3_ added to the right)


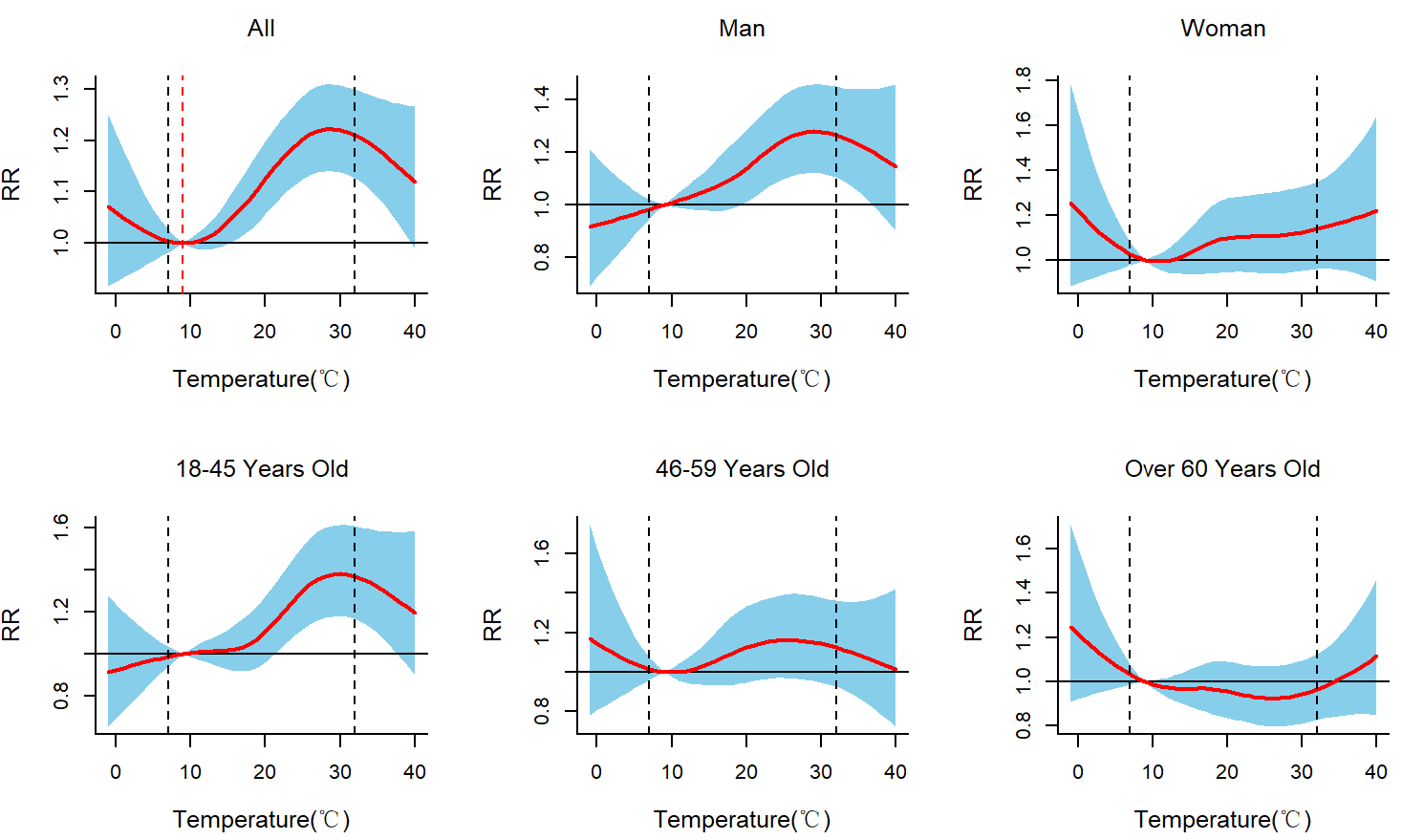


Figure S2 Response association between the overall cumulative exposure and the associated temperature distribution for different populations of IEADs in the main urban area of Chongqing


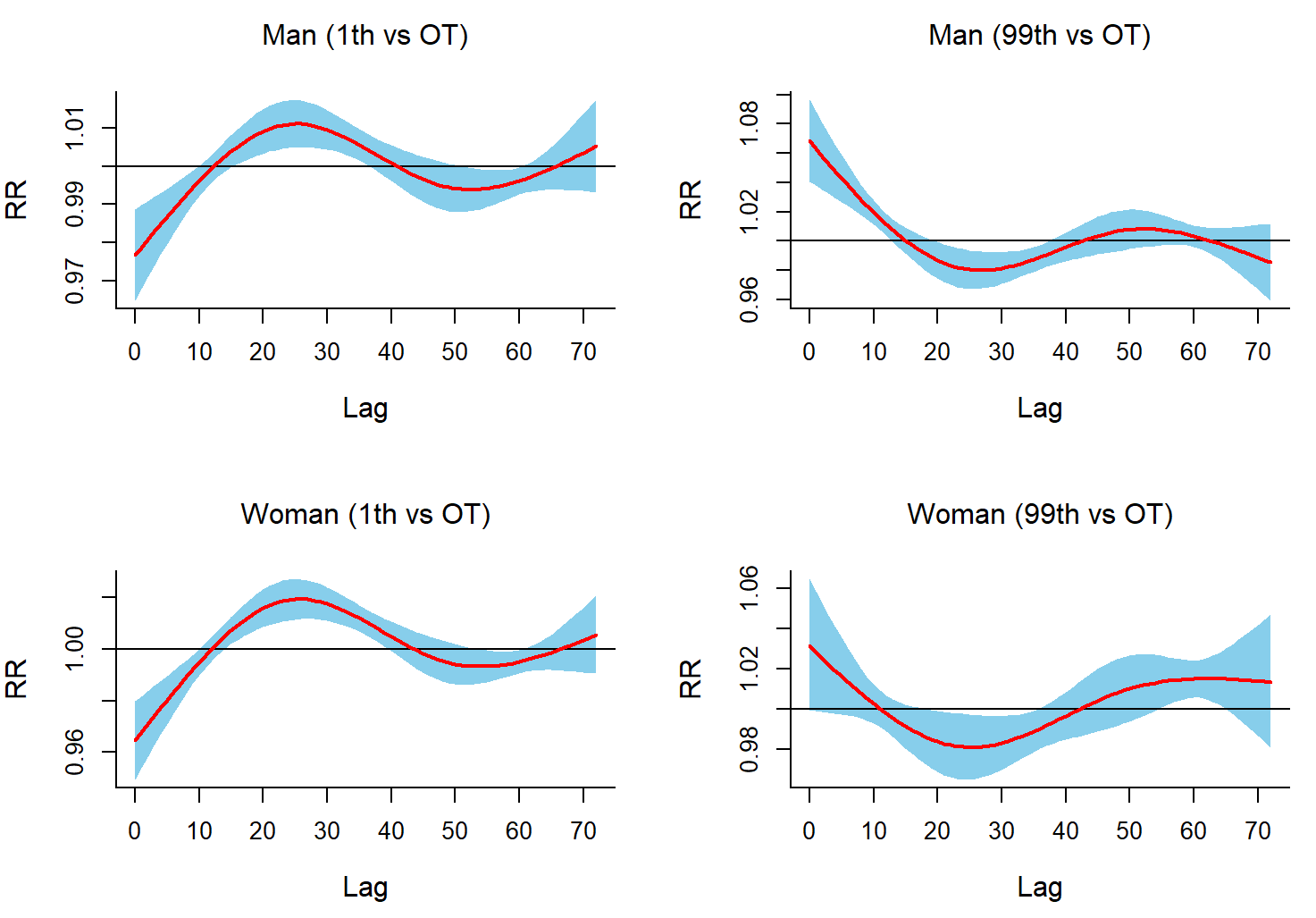


Figure S3 Lagged response association between extreme low temperature (5℃: 1st percentile temperature) and extreme high temperature (36℃: 99th percentile temperature) in different gender.


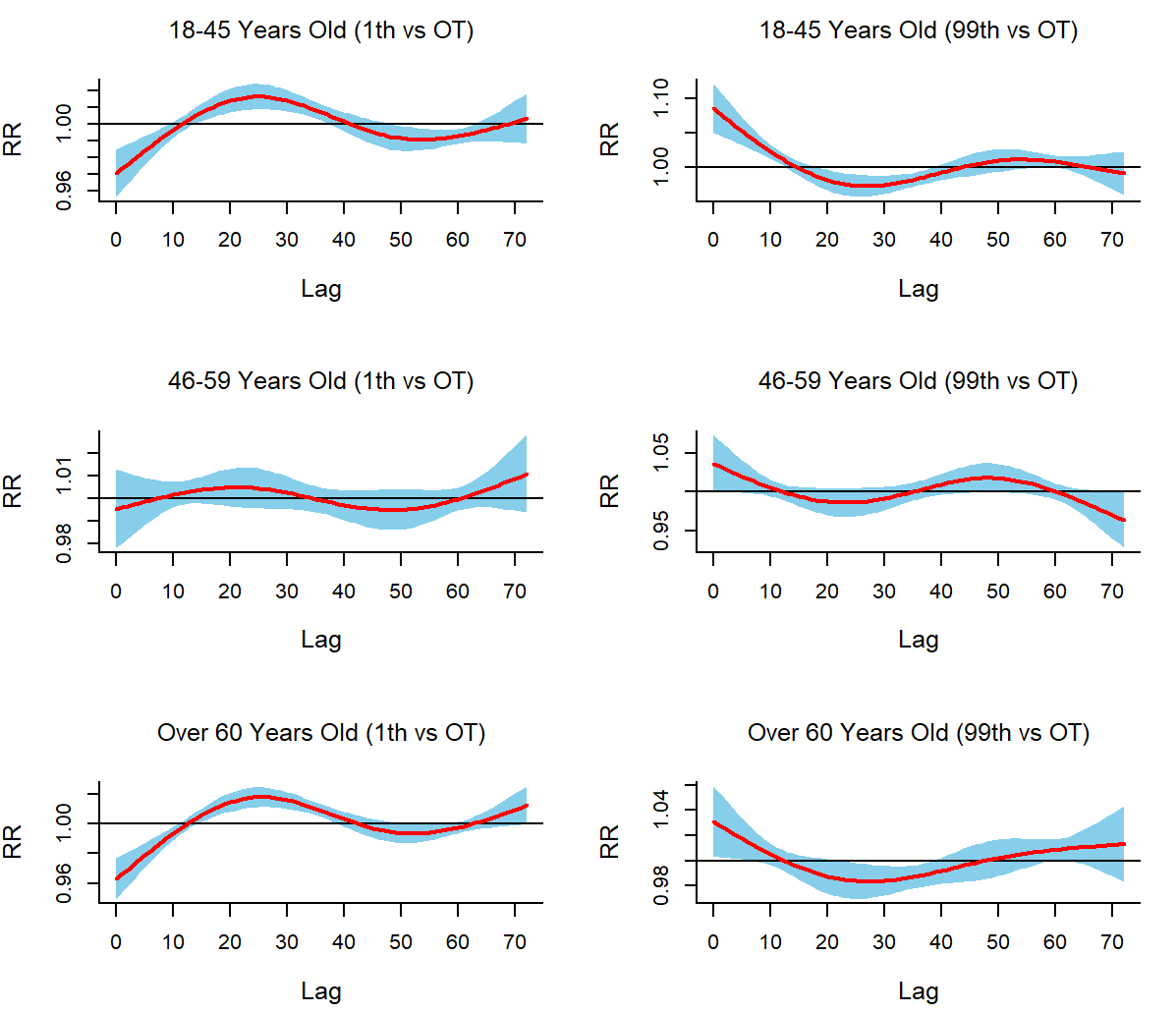


Figure S4 Lagged response association between extreme low temperature (5℃: 1st percentile temperature) and extreme high temperature (36℃: 99th percentile temperature) in different age groups.
